# Association between novel arterial stiffness indices and risk factors of cardiovascular disease

**DOI:** 10.1186/s12872-016-0389-x

**Published:** 2016-11-07

**Authors:** Masaki Okamoto, Fumiaki Nakamura, Terunaga Musha, Yasuki Kobayashi

**Affiliations:** 1 Department of Public Health, Graduate School of Medicine, The University of Tokyo, 7-3-1 Hongo, Bunkyo-ku, Tokyo, 113-0033 Japan; 2 Hachinohe West Health Medical Plaza, 74-1, Nakatsubo, Naganawashiro, Hachinohe, Aomori 039-1103 Japan

**Keywords:** Arterial stiffness, Index, Noninvasive, Clinical validity, Prevalence, General population

## Abstract

**Background:**

Prevention and early detection of arterial stiffness are required to avoid severe cardiovascular events. Recently, new noninvasive arterial stiffness indices, the arterial pressure volume index (API) and the arterial velocity pulse index (AVI), have been developed. The purpose of this study was to examine the clinical validity of these new indices by investigating the association between known risk factors of cardiovascular disease (CVD) and API or AVI in a large population.

**Methods:**

This cross-sectional survey included 7248 adults who underwent an annual medical checkup at a single medical institution. API and AVI were measured using cuff oscillometry by trained nurses. We used correlation coefficients, t-tests, and multiple regression analyses to evaluate associations, and calculated intraclass correlation coefficients (ICC) to examine test-retest reliabilities of these indices.

**Results:**

Mean age was 45.5 years (SD = 5.8), and 4083 (56.3 %) participants were men, while 3165 were women. Mean values of API and AVI were 25.1 (SD = 7.0) and 16.6 (SD = 5.4), respectively. API was strongly correlated with body mass index (BMI), systolic blood pressure (sBP), and diastolic blood pressure (dBP) (*r* > 0.3, *p* < 0.001). AVI was strongly correlated with age, sBP, and API (*r* > 0.3, *p* < 0.001). Multiple regression analyses showed that sex, age, BMI, and sBP were independently associated with API. Sex, age, BMI, sBP, fasting plasma glucose (FPG), and smoking condition were also independently associated with AVI. As reliabilities of measurements, the ICC of API was 0.74, and the ICC of AVI was 0.80.

**Conclusions:**

These new noninvasive arterial stiffness indices, which had high test-retest reliabilities, were associated with known risk factors of CVD. Further study is warranted to determine the clinical validity of these indices.

## Background

Arterial stiffness is independently associated with an increased risk of cardiovascular disease (CVD) [1, 2]. Several studies have shown that pulse wave velocity (PWV), a noninvasive clinical index of arterial stiffness, predicts cardiovascular events and all-cause mortality [3–8]. Prevention and early detection of arterial stiffness need to be promoted to avoid severe cardiovascular events. Existing methods of assessing arterial stiffness or atherosclerosis such as PWV, Cardio Ankle Vascular Index (CAVI), or carotid artery intima-media thickness (IMT) are technically difficult for medical staff unfamiliar with such measurements. Moreover, these instruments are uncomfortable for patients, because of long measurement times and postural requirements. Simpler and easier methods that can be used in daily clinical settings are required. Recently, new novel arterial stiffness indices have been developed, including the arterial pressure volume index (API), and the arterial velocity pulse index (AVI). API and AVI are measured oscillometrically at one-side of the upper arm, and in a sitting position similar to conventional measurements of blood pressure.

While technical theories support the utility of API and AVI as arterial stiffness indices [9, 10], the clinical significance of the indices has not been fully investigated, especially in the general healthy population. Thus, the aim of this study was to examine the clinical validity of the new indices by investigating associations between known risk factors for CVD and API or AVI, and also to examine test-retest reliabilities of the indices in a large healthy population.

## Methods

### Study participants

We conducted a cross-sectional survey of 7248 healthy adults (4083 males and 3165 females) who were aged 20 and over, and who underwent an annual medical checkup at a single large medical institution in Hachinohe, Aomori prefecture, Japan, between April 2014 and March 2015. The purpose and procedure of the study were explained to participants, who then provided written informed consent to be included.

The study protocol was approved by the Ethics Committee at Hachinohe West Health Medical Plaza and the Ethics Committee at The University of Tokyo Graduate School of Medicine (approval number: 10588).

### API and AVI

Using the time series of occlusive cuff pressure and amplitudes of pulse oscillations, local slopes of the curve between decreasing cuff pressure and corresponding arterial pressure volume were calculated [9]. A complete pressure-volume curve was derived from numerical integration of the local slopes. The curve was fitted using an equation: F(x) = A arctan(Bx + C) + D (A,C,D; constant),and API was defined as a numerical coefficient B of the equation, which could evaluate arterial stiffness as it closely reflected the slope of the curve. AVI has the characteristic of pulse waves at higher cuff pressures compared with systolic BP, and central BP is simultaneously reflected in pulse waves [10–12]. The systolic latter oscillometric waveform was increased by the enhancement of reflected wave, which was influenced by aging and peripheral arterial resistance, and then it steeply descended. On the other hand, the incident waveform was not influenced by reflected wave. Therefore, a ratio of these amplitude of differentiated waveform was defined as AVI, which indicated the reflected wave magnitude. Increased AVI indicated enhancement of reflected waves, which was considered to be influenced by aging or advanced arteriosclerosis.

The indices were measured using cuff oscillometry with PASESA AVE-1500 (Shisei Datum, Tokyo, Japan) by trained nurses. A cuff was wrapped around one-side of the upper arm of seated participants after they had an adequate resting period. One minute was needed for measurements conducted during the series of medical checkups for study participants.

### Reliability of measurements

Participants included for the first two months (*n* = 1085) were measured twice within an interval of 5 min to examine the reliability of measurements.

### Other variables

Individual results of medical checkup for study participants were also collected with their consent. Information about age, sex, former diagnosis of major diseases, medication history, family history of hypertension, smoking condition, alcohol consumption, and exercise habits was obtained using a self-administered questionnaire. Height, weight, abdominal circumference, blood pressure, and heart rate were measured by nurses. Blood tests, including hemoglobin, total protein, albumin, eGFR, fasting plasma glucose (FPG), and cholesterol were also conducted for each participant.

### Other arterial stiffness or atherosclerosis indices

IMT, CAVI, and Ankle Brachial pressure Index (ABI) were also conducted in some of the participants (IMT, *n* = 513; CAVI and ABI, *n* = 121). These participants voluntarily requested these tests related to arterial stiffness or atherosclerosis.

### Statistical analysis

We calculated correlation coefficients between API or AVI and for continuous variables. We used t-tests to compare differences in mean API or AVI according to dichotomous variables. Regarding reliabilities of measurements, we calculated intraclass correlation coefficients (ICC) of API and AVI among those who were measured twice in the first two months (*n* = 1085). Finally, multiple regression analysis was performed to investigate the association between API or AVI and other variables, using a backward stepwise elimination. A *p* value of < 0.05 was considered statistically significant. All analyses were performed using Stata/MP ver. 13.1 (StataCorp, TX, USA).

## Results

**Fig. 1:**
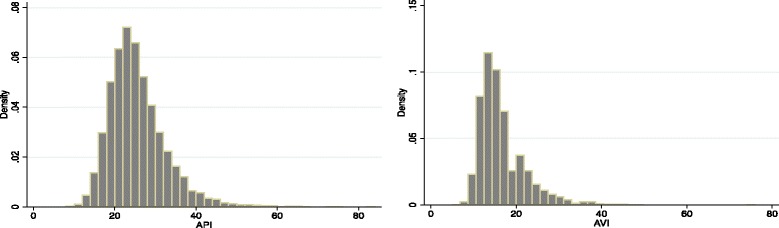
Histogram of distribution of API and AVI values

Table 1 shows baseline characteristics of the study participants. Mean age was 45.5 years (SD = 5.8), and 4083 (56.3 %) participants were men, while 3165 were women. With regard to average values of body mass index (BMI), BP, and blood tests, participants did not exhibit a skewed distribution to the normal range. The proportion of participants who had family history of hypertension was 30.9 %, whereas 9.2 % of participants were diagnosed with hypertension. Overall, 2531 (34.9 %) participants were current smokers. Mean API was 25.1 (SD = 7.0), and mean AVI was 16.6 (SD = 5.4). The distributions of API and AVI are shown in Fig. 1. For reliabilities of measurements, the ICC of API was 0.74, and the ICC of AVI was 0.80.

**Table 1:** Baseline characteristics of participants

No. of subjects	7248	
Sex		
Male	4083	[56.3]
Female	3165	[43.7]
Age (years)	45.5 ± 5.8	
Height (cm)	165.1 ± 8.5	
BMI (kg/m^2^)	23.8 ± 4.1	
Abdominal circumference (cm)	82.7 ± 10.5	
sBP (mmHg)	120.5 ± 15.8	
dBP (mmHg)	77.5 ± 10.7	
HR (/min)	66.1 ± 9.4	
Hemoglobin (g/dL)	14.5 ± 1.8	
Total protein (g/dL)	7.1 ± 0.4	
Albumin (g/dL)	4.4 ± 0.2	
eGFR (%)	76.6 ± 12.5	
AST (IU/L)	21.8 ± 12.3	
ALT (IU/L)	24.9 ± 20.0	
FPG (mg/dL)	103.0 ± 20.6	
HbA1c (NGSP, %)	5.5 ± 0.7	
Total cholesterol (mg/dL)	203.2 ± 34.2	
LDL cholesterol (mg/dL)	125.1 ± 33.6	
HDL cholesterol (mg/dL)	65.4 ± 17.8	
Triglyceride (mg/dL)	117.3 ± 108.6	
Hypertension	668	[9.2]
Diabetes mellitus	286	[3.9]
Dyslipidemia	393	[5.4]
Medications		
Antihypertension	634	[8.7]
Antidiabetes	219	[3.0]
Lipid-lowering	346	[4.7]
Family history of hypertension	2242	[30.9]
Current smoker	2531	[34.9]
API	25.1 ± 7.0	
AVI	16.6 ± 5.4	

Data are means ± SD or *n* [%]. *Abbreviations*: *BMI* body mass index, *sBP* systolic blood pressure, *dBP* diastolic BP, *HR* heart rate, *eGFR* estimated glomerular filtration rate, *AST* aspartate aminotransferase, *ALT* alanine aminotransferase, *FPG* fasting plasma glucose, *HbA1c* hemoglobin A1c, *LDL* low density lipoprotein, *HDL* high density lipoprotein, *SD* standard deviation

**Fig. 2:**
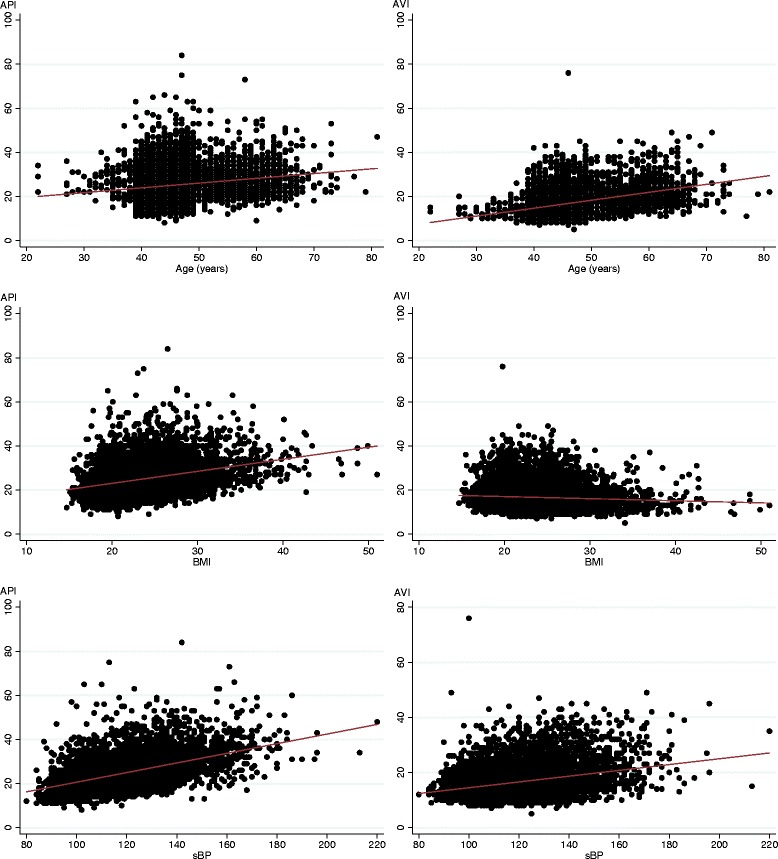
Correlation between API or AVI and age, BMI, sBP

Table 2 shows correlation coefficients between API or AVI and continuous variables. Both API and AVI were associated with several variables. Particularly, API was strongly correlated with BMI, sBP, and dBP (*r* > 0.3, *p* < 0.001). AVI was strongly correlated with age, sBP, and API (*r* > 0.3, *p* < 0.001). Scatter plots of API and AVI by age, BMI, and sBP are shown in Fig. 2.

**Table 2:** Coefficients of correlation between API or AVI and other variables

Variables	Male		Female		All participants	
	API	AVI	API	AVI	API	AVI
API		0.232✳✳		0.383✳✳		0.316✳✳
Age (years)	0.143✳✳	0.450✳✳	0.244✳✳	0.355✳✳	0.180✳✳	0.387✳✳
Height (cm)	-0.057✳✳	-0.161✳✳	-0.106✳✳	-0.188✳✳	-0.080✳✳	-0.220✳✳
BMI (kg/m^2^)	0.303✳✳	-0.072✳✳	0.363✳✳	-0.013	0.318✳✳	-0.071✳✳
Abdominal circumference (cm)	0.281✳✳	-0.045✳	0.334✳✳	-0.004	0.285✳✳	-0.061✳✳
sBP (mmHg)	0.516✳✳	0.315✳✳	0.538✳✳	0.415✳✳	0.493✳✳	0.306✳✳
dBP (mmHg)	0.371✳✳	0.265✳✳	0.381✳✳	0.371✳✳	0.346✳✳	0.257✳✳
HR (/min)	0.152✳✳	0.129✳✳	0.136✳✳	0.025	0.144✳✳	0.087✳✳
Hemoglobin (g/dL)	0.081✳✳	-0.051✳	0.015	0.009	0.002	-0.122✳✳
Total protein (g/dL)	0.129✳✳	-0.066✳	0.060	0.009	0.091✳✳	-0.043
Albumin (g/dL)	0.073✳	-0.188✳✳	0.009	0.011	0.033	-0.129✳✳
eGFR (%)	-0.034	-0.035	0.065✳	0.055✳	0.018	0.020
AST (IU/L)	0.097✳✳	0.029	0.106✳✳	0.060✳	0.080✳✳	-0.001
ALT (IU/L)	0.136✳✳	-0.054✳	0.141✳✳	0.025	0.106✳✳	-0.076✳✳
FPG (mg/dL)	0.150✳✳	0.190✳✳	0.213✳✳	0.120✳✳	0.147✳✳	0.112✳✳
HbA1c (NGSP, %)	0.141✳✳	0.172✳✳	0.202✳✳	0.147✳✳	0.154✳✳	0.142✳✳
Total cholesterol (mg/dL)	0.046✳	-0.016	0.086✳✳	0.086✳✳	0.058✳✳	0.010
LDL cholesterol (mg/dL)	0.040✳	-0.053✳✳	0.114✳✳	0.043✳	0.067✳✳	-0.036✳
HDL cholesterol (mg/dL)	-0.034✳	0.051✳	-0.121✳✳	0.024	-0.061✳✳	0.085✳✳
Triglyceride (mg/dL)	0.079✳✳	0.033	0.193✳✳	0.125✳✳	0.082✳✳	0.007

✳✳*p* < 0.001

✳*p* < 0.05

Table 3 shows differences in API or AVI by dichotomous variables, using t-tests. Mean API and AVI in women were higher compared with men. Mean API and AVI in those with hypertension, dyslipidemia, diabetes, or family history of hypertension were higher compared with those without. Mean AVI in everyday drinkers was also higher, but not API.

**Table 3:** Differences in API or AVI by dichotomous variables, using t-tests

	Mean API (± SD)		*P* value	Mean AVI		*P* value
	+	-		+	-	
Male	24.8 ± 6.1	25.3 ± 8.0	0.0035	15.9 ± 4.9	17.5 ± 5.9	<0.001
Hypertension	27.5 ± 7.0	24.6 ± 6.8	<0.001	18.5 ± 6.4	16.2 ± 5.1	<0.001
Dyslipidemia	26.8 ± 6.8	24.7 ± 6.9	<0.001	17.5 ± 6.0	16.4 ± 5.2	<0.001
Diabetes	27.3 ± 6.5	24.8 ± 6.9	<0.001	18.6 ± 6.1	16.3 ± 5.2	<0.001
Family history of hypertension	25.6 ± 7.2	24.8 ± 6.8	<0.001	16.9 ± 5.5	16.5 ± 5.4	0.0044
Current smoker	24.4 ± 6.3	25.1 ± 7.2	<0.001	16.3 ± 5.0	16.5 ± 5.4	0.0779
Everyday drinking	24.9 ± 6.4	24.8 ± 7.0	0.5629	16.8 ± 5.4	16.3 ± 5.2	<0.001
Exercise habit	25.0 ± 6.7	24.8 ± 6.9	0.5691	16.3 ± 5.2	16.5 ± 5.3	0.2939

Table 4 shows the results of multiple regression analysis of API or AVI using the backward stepwise method. Sex, age, BMI, and sBP, were independently associated with API. Sex, age, BMI, sBP, FPG, and smoking condition were also independently associated with AVI.

**Table 4:** Multiple regression analysis of API or AVI (backward stepwise)

	API				AVI			
	coef.	95 % CI	*P* value	p.c.	coef.	95 % CI	*P* value	p.c.
Male	-2.921	-3.218, -2.623	<0.001	-0.215	-2.752	-3.001, -2.504	<0.001	-0.262
Age	0.074	0.047, 0.102	<0.001	0.068	0.296	0.274, 0.318	<0.001	0.318
BMI	0.313	0.275, 0.351	<0.001	0.194	-0.233	-0.265, -0.202	<0.001	-0.180
sBP	0.206	0.196, 0.216	<0.001	0.444	0.119	0.111, 0.127	<0.001	0.344
FPG		Removing			0.015	0.008, 0.021	<0.001	0.058
LDL-c		Removing			-0.003	-0.007, 0.000	0.067	-0.023
Smoker		Removing			0.807	0.569, 1.045	<0.001	0.083
adjusted R-square = 0.297					adjusted R-square = 0.267			

*Abbreviations*: *coef*. coefficient, *p.c.* partial correlation

Finally, Table 5 shows correlation coefficients between API or AVI and previous arterial stiffness or atherosclerosis indices. Except for CAVI-API, statistically significant associations were found for all indices.

**Table 5:** Correlation coefficients between API or AVI and IMT, ABI, CAVI

	API		AVI	
	r	*P* value	r	*P* value
IMT	0.116	<0.01	0.210	<0.01
ABI	-0.229	<0.01	-0.259	<0.01
CAVI	0.144	0.115	0.200	<0.05

*Abbreviations*: *IMT* intima-media thickness, *ABI* ankle brachial pressure index, *CAVI* cardio ankle vascular index

## Discussion

In this study, we found significant associations between API or AVI and known risk factors for CVD, which might support the clinical validity of these new arterial stiffness indices. In addition, the test-retest reliabilities of API and AVI were acceptably high. This is the first study to assess associations between known risk factors for CVD and API or AVI in a large healthy population. Typical risk factors such as sex, age, BMI, and sBP were independently associated with API as determinants. Similarly, AVI was independently associated with sex, age, BMI, sBP, FPG, and smoking condition. This suggests that the indices could be useful total vascular risk markers.

Regarding test-retest reliability of the measurements, the ICCs of API (0.74) and AVI (0.80) were acceptably high, because the indices were affected by enduring hemodynamic changes, even after a 5-minute interval.

API and AVI were associated with typical risk factors for arterial stiffness such as age, sex, blood pressure, diseases (hypertension, dyslipidemia, diabetes), and family history of hypertension. Other arterial stiffness indices such as PWV or CAVI evaluate vascular condition, especially reduction in arterial compliance along the long axis of arteries, by attaching two or more cuffs. IMT assesses structural changes in arteries. API and AVI evaluate arterial compliance similarly; however, these indices focus the pulse wave component, extracted with a cuff attached, at only one place. This study showed that API significantly correlated with IMT and CAVI. In addition, AVI significantly correlated with IMT, ABI, and CAVI. Despite differences in technical theory of the measurements, the findings above support the validity of API and AVI as vascular risk markers.

Although, in univariate analysis, mean API and AVI in current smokers were lower compared with non-smokers, this relationship disappeared in multiple regression analysis. Confounders such as sex or age might affect this association. Furthermore, items related to API or AVI were different between the indices. This might indicate differences in vascular sites evaluated based on the background technical theory. Specifically, API mainly reflects stiffness of the peripheral arteries, whereas AVI reflects stiffness of the central arteries [9, 10]. Compared with API, AVI includes pulse wave reflected central blood pressure as a component. Indeed, some studies showed that AVI was an independent predictor of central blood pressure in patients with cardiovascular disease [11, 12]. We found that moderate or strong correlations between API and BMI, sBP, dBP, and between AVI and age, sBP, dBP existed while that modest but statistically significant correlations existed between these arterial stiffness indices and other factors. We can say that both of API and AVI are obviously BP-dependent indices as this study showed. However, BPs were not directly included as part of calculation as mentioned above, and therefore, the association was due to factors related arterial stiffness but not mathematical reasons. The same applied for BMI and age. These observations deserve further investigation.

Some limitations of the present study must be acknowledged. First, self-report measurements are generally less accurate; therefore, past history or family history of diseases might be biased. However, the prevalences of family history of each disease were consistent with available Japanese data (13). Second, participants in the current study were not necessarily representative of the general Japanese population. Participants in this study were individuals who had a medical checkup of their own volition at a large-scale medical facility. While a large number of community residents visit the facility, study participants were not chosen at random. However, this population comprised people mainly in their 40s, with the prevalence of cardiovascular risk factors similar to available Japanese data [13]. Third, other unmeasured factors such as socioeconomic factors may have affected our findings. Social structure and educational background in the catchment area of the current study is somewhat different from typical urban areas, because the number of residents engaged in primary industry is about 1.6 times the average in Japan [14]. Fourth, we could not compare new arterial stiffness indices with traditional and standard ones such as carotid-femoral PWV because of the study design. Finally, the cross-sectional nature of this study limited our ability to discuss causality.

In the future, a longitudinal study to determine the extent of the effect that measuring API and AVI has on the development of cardiovascular diseases, and all-cause mortality, should be conducted. Because API and AVI are measured non-invasively in a short time (about 1 minute), similar to conventional measurement of blood pressure, the indices could allow people easy access to vascular condition information, not only in medical checkups, but also in daily life settings, without any need for medical technicians. This study suggests that API and AVI might have clinical significance as arterial stiffness measures. Thus, further research into API and AVI in terms of arterial stiffness prevention is warranted.

## Conclusion

These new noninvasive arterial stiffness indices, which had high test-retest reliabilities, were associated with known risk factors for CVD. Further study is warranted to determine the clinical validity of these indices.

## Abbreviations

ABI: ankle brachial pressure index
API: arterial pressure volume index
AVI: arterial velocity pulse index
BMI: body mass index
CAVI: cardio ankle vascular index
CVD: cardiovascular disease
dBP: diastolic blood pressure
FPG: fasting plasma glucose
ICC: intraclass correlation coefficient
IMT: intima-media thickness
PWV: pulse wave velocity
sBP: systolic blood pressure
